# Structural and Antigenic Definition of Hepatitis C Virus E2 Glycoprotein Epitopes Targeted by Monoclonal Antibodies

**DOI:** 10.1155/2013/450963

**Published:** 2013-07-09

**Authors:** Giuseppe Sautto, Alexander W. Tarr, Nicasio Mancini, Massimo Clementi

**Affiliations:** ^1^Laboratorio di Microbiologia e Virologia, Università Vita-Salute San Raffaele, Via Olgettina 58, 20132 Milano, Italy; ^2^School of Molecular Medical Sciences, The University of Nottingham, Nottingham NG7 2UH, UK; ^3^Biomedical Research Unit in Gastrointestinal and Liver Diseases, The University of Nottingham, Nottingham NG7 2UH, UK

## Abstract

Hepatitis C virus (HCV) is the major cause of chronic liver disease as well as the major indication for liver transplantation worldwide. Current standard of care is not completely effective, not administrable in grafted patients, and burdened by several side effects. This incomplete effectiveness is mainly due to the high propensity of the virus to continually mutate under the selective pressure exerted by the host immune response as well as currently administered antiviral drugs. The E2 envelope surface glycoprotein of HCV (HCV/E2) is the main target of the host humoral immune response and for this reason one of the major variable viral proteins. However, broadly cross-neutralizing monoclonal antibodies (mAbs) directed against HCV/E2 represent a promising tool for the study of virus-host interplay as well as for the development of effective prophylactic and therapeutic approaches. In the last few years many anti-HCV/E2 mAbs have been evaluated in preclinical and clinical trials as possible candidate antivirals, particularly for administration in pre- and post-transplant settings. In this review we summarize the antigenic and structural characteristics of HCV/E2 determined through the use of anti-HCV/E2 mAbs, which, given the absence of a crystal structure of this glycoprotein, represent currently the best tool available.

## 1. Introduction: Functions of HCV Glycoproteins

The hepatitis C virus (HCV) glycoproteins E1 and E2 are the most important targets of neutralizing antibodies (Abs). This is a direct consequence of their roles in mediating entry of the virus into susceptible cells in a pH- and clathrin-dependent manner [1–5]. The two genes encoding the HCV glycoproteins lie in the N-terminal part of the HCV genome. The glycoproteins are expressed initially as part of the virus polyprotein, with the mature proteins being released by the action of the host cellular proteinases signal peptidase and signal peptide peptidase [6]. Depending on virus isolate, mature, cleaved E1 protein possesses 192 amino acids and E2 between 363 and 369 amino acids. The glycoproteins form heterodimers through interactions between their transmembrane domains, each chaperoning the folding of the other during synthesis [7, 8]. Amino acid variation in E1 and E2 proteins exceeds 37% among infectious primary isolates, highlighting the extreme genetic diversity that is tolerated in the E1 and E2 genes [9]. The greatest amino acid variation is observed in three hypervariable regions (HVRs) in the E2 protein [10–12]. HVR1 is a 26-27 amino acid region at the extreme N-terminus of E2 and displays the greatest variability in the HCV polyprotein. HVR2 is proximal to the CD81 binding regions of E2, while the intergenotypic variable region (IgVR) lies closer to the transmembrane domain of E2 [12]. Despite this heterogeneity, both proteins possess conserved N- and O-linked glycans, and there is evidence of extensive glycosylation on the surface of both proteins [13, 14]. E2 is the major receptor binding protein, interacting with cell surface molecules CD81, SR-BI, and occludin [15–17]. The interaction between E2 and cell surface receptors has been well described: the binding surface with CD81 is a discontinuous surface encompassing three highly conserved regions of the E2 protein [18–20], while the interaction with SR-BI is believed to be mediated by the N-terminal hypervariable region (HVR1) of the E2 protein [21]. Blockade of receptor interactions is likely to be the main action of neutralizing Abs.

## 2. Structure of E1 and E2

There is little direct evidence for the structure and domain architecture of the E1 and E2 glycoproteins. Efforts to crystalize these proteins have so far yielded no accurate structure. As a result, attempts to assign domain structures to these proteins have used a combination of computational models [22] and biochemical analyses [23]. Early studies of the structure of E2 mapped the HCV primary amino acid sequence onto the crystal structure of a prototype *Flavivirus* E protein possessing a type II fusion protein architecture [22]. Recent analyses have mapped the disulphide bridging patterns within the ectodomain of E2, finding patterns consistent with a type II fusion protein [23]. In this study, three domains were defined, relating to the three classical domains of *Flavivirus* glycoproteins, DI, DII, and DIII. In this model DI is discontinuous and features an immunoglobulin fold including the CD81 binding site. DII is predicted to possess a hydrophobic fusion peptide. However, members of the genera *Hepacivirus* and *Flavivirus* are only remotely related. Studies describing the crystal structure of the Pestivirus E2 protein have queried the validity of assigning a type II classification to the HCV glycoprotein [24, 25]. The Pestivirus protein possesses a previously undescribed four domain structure, with four contiguous domains. While this class of structure cannot be assigned to HCV/E2 yet, this data illustrates the possibility that the HCV glycoprotein might belong to an as-yet undescribed class of fusion proteins.

The structure of E1 has been even more intractable. Expressed in the absence of E2, E1 aggregates and does not fold correctly, making structural analysis impossible [26, 27]. This is consistent with reports that E1 might contain the hydrophobic peptide required for envelope fusion [28, 29]. In the absence of direct structural evidence, many studies have investigated the antigenic structure of the E1 and E2 proteins to elucidate its architecture.

## 3. The Antigenic Structure of HCV/E2 Glycoprotein

There is a wealth of data describing the role of neutralizing Abs in protecting against HCV infection, which has recently been the focus of reviews by Edwards et al. [30] and Fafi-Kremer et al. [31]. Here we will focus on the binding of Abs to specific epitopes in HCV/E2 and the different properties of Abs targeting these epitopes.

Glycoprotein E2 appears to be the more immunogenic of the two HCV glycoproteins [53]. Studies isolating monoclonal antibodies (mAbs) from HCV-infected individuals and experimentally immunized animals have predominantly isolated anti-E2 Abs. Epitopes can be classified as either linear (recognizing linear peptides with contact residues that are only a few residues apart) or conformation sensitive (with contact residues distantly distributed along the primary amino acid sequence but proximal in the three-dimensional space occupied by the protein). Many of the Abs elicited by immunization with recombinant forms of the glycoproteins recognize linear epitopes [32, 54]. In contrast, mAbs isolated from infected humans more often recognize conformation-sensitive epitopes [43, 46, 55] (Tables 1 and 2). Thus the nature of the immunogen is critical to the quality of the Ab response produced. This is also reflected in the neutralizing capacity of mAbs isolated by the two approaches, while many human mAbs possess neutralizing potency [44, 56–58], murine mAbs recognizing linear epitopes have restricted specificity and no neutralizing properties [3].

Initial attempts to map murine mAbs utilized overlapping panels of linear peptides designed from reference isolates [32]. This identified a range of epitopes that were accessible on recombinant proteins and virus-like particles across both E1 and E2 that were immunogenic when animals were immunized with recombinant proteins [54]. With some notable exceptions, mAbs recognizing linear peptides demonstrated restricted patterns of recognition and neutralization. This was particularly notable for the mAbs recognizing epitopes in HVR1 but also for other Abs recognizing linear epitopes [3] (Table 2).

### 3.1. The HVR1 as a Target for Abs

Following early studies of the Ab response to HCV, the HVR1 was thought to be the major immunodominant neutralization region in the E2 protein. Abs directed to HVR1 were isolated from infected chimpanzees and protected against infection [59]. This was supported by indirect evidence that sequence variability in the HVR1 during chronic infection corresponded to selection of particular HVR1 variants, suggesting that Ab neutralization drives selection of HVR1 quasispecies [60]. HVR1 was subsequently identified to have a direct role in entry, binding to SR-BI and augmenting infectivity [17, 21, 61, 62]. HVR1 also modulates neutralization by Abs targeting the conserved CD81 binding site [63], suggesting that the observed genetic heterogeneity might contribute to persistence in the presence of host broadly neutralizing Abs. HVR1 acts as an immune decoy and prevents generation of a protective Ab response to conserved epitopes in regions essential for E2 function [63]. Mapping has revealed multiple epitopes in the HVR1 region, all of which are restricted in their reactivity. These Abs have different properties. Abs targeting the very N-terminus of E2 have no neutralizing activity [3]. In contrast the rat mAb 9/27 inhibits binding of E2 to SR-BI and potently neutralizes infectivity of genotype 1a strains [3, 64]. This mAb mapped to an epitope in the C-terminal half of the 27-amino acid HVR1 as did other HVR1-specific Abs that neutralized infection [65]. More recently, screening of large panels of mAbs generated by immunization of mice has identified neutralizing Abs with epitopes in the C-terminal end of HVR1 (J6.36, J6.103, and H77.16), which inhibit E2 interaction with SR-BI (Table 1) [38]. These studies highlight not only the potential for anti-HVR1 Abs for preventing infection but also the limitations of restricted reactivity due to sequence variation in primary HCV isolates across the linear epitopes in this region, as recently confirmed in phase 1B clinical trials [66]. 

### 3.2. Epitopes in the 412–423 Amino Acid Region (Epitope I)

Immediately downstream of the HVR1 is a highly conserved region of the HCV genome. Early studies identified murine mAbs that bound to this region in proteins representing genetically diverse HCV strains [28, 32, 54]. This region was established to play an important role in forming complexes of E2 with CD81 [28] and to have a direct role in entry by mediating CD81 binding [19]. Murine mAbs (AP33 and 3/11) that recognized a peptide defined as amino acids 412–423 (also known as epitope I) of the HCV polyprotein were found to efficiently neutralize entry (Table 1) [3, 34]. Although these Abs were originally thought to recognize the same conserved epitope, molecular dissection of their respective contact residues revealed that they recognize overlapping epitopes with different neutralizing potentials [35]. Specifically, AP33 possessed very high neutralizing potency, binding to contact residues at positions L413, N415, G418, and W420. In contrast, mAb 3/11 formed interactions with residues N415, W420, and H421 and neutralized the same panel of viruses with lower potency [35]. The interaction of AP33 with its epitope was subsequently confirmed by solving the crystal structure of the E2 peptide in complex with AP33 [67, 68]. Other mAbs recognizing this region have more recently been described, including human mAb HCV1 [39], which binds predominantly at positions L413 and W420 [69]. Isolation of mouse Abs using a neutralization screening procedure also isolated a broadly neutralizing mAb, H77.39, that recognized this region [38]. Screening this mAb with a random mutant E2 library resulted in residues N415 and N417 as important in recognition. Indeed, of all 78 anti-E2 mAbs screened in this assay, this was by far the most potent at neutralizing infection. As well as blockading interaction with CD81, H77.39 also inhibited SR-BI binding to E2, suggesting that the proximity of this mAb binding to the HVR1 resulted in two combined modes of neutralizing action. Together these data confirm that discrete, overlapping neutralization epitopes exist in this highly conserved region of E2. Interestingly, all mAbs so far analyzed require W420 as a contact residue. This residue has been described to be critically important in binding to CD81 [19], making up one of the discontinuous regions of the conformation sensitive CD81 binding site [70]. Blockade of the interaction with CD81 is the likely mode of neutralization of these Abs, and it is clear that this region of the protein plays a critical role in the entry pathway of HCV.

Studies of the prevalence of Abs directed to this region in chronically infected individuals have revealed a very low seroprevalence [37, 71], suggesting that this region is not naturally immunogenic. Importantly, while around 2.5% of chronically infected individuals raised an Ab response to the region, less than 1% of infections resulted in an Ab response containing Abs sharing epitope specificity with AP33 [71]. Abs directly purified from those patients with a detectable response to the region spanning 412–423 amino acids were able to broadly and potently neutralize infection, highlighting the potency of Abs to this region. In an alternative approach, Abs purified from hyper-immune globulin by reactivity to a peptide corresponding to the 412–419 amino acid region efficiently neutralized entry, demonstrating that epitopes without the conserved tryptophan at amino acid 420 also contribute to neutralization. Together these studies demonstrate that the Abs to this region have potential for therapeutic administration and vaccine design.

### 3.3. The Discontinuous CD81 Binding Region as a Target for Abs

While locating linear epitopes recognized by mAbs, such as AP33, is a relatively straightforward process using overlapping peptides, identification of the contact residues of conformation-sensitive Abs targeting the discontinuous CD81 binding site requires a combination of techniques. We and others have mapped a range of Abs directed to the HCV glycoproteins, using a combination of single residue alanine-scanning mutagenesis, mimotope affinity selection from random phage-displayed peptide libraries, mapping using random yeast-display libraries, and competition analysis with mAbs recognizing linear peptides [38, 44–46, 49, 52]. These analyses identified the discontinuous CD81 binding site as a key conserved neutralization determinant. Analysis of the neutralizing potential of Abs against all six major genotypes of HCV highlighted key conserved amino acids that were common to the interaction with CD81 and neutralizing mAbs. One important region of E2 that is involved in this interaction was described to be between amino acids 523 and 535 [19]. Many of the broadly neutralizing human anti-E2 mAbs recognize one of a small number of conserved residues in this region, including G523, Y527, W529, G530, and D535 (Table 1) [44–46, 49, 52]. However, similarly to mAbs directed to the linear region 412–423, different mAbs possess slightly different contact residues. This region is consistent with “Antigenic Domain B” defined by Keck and colleagues and “Antigenic Region 3” (AR3) as defined by Law et al. [46, 72]. This region appears to be immunogenic in natural infection, as human mAbs to overlapping epitopes in this region have been isolated from independent patients, using affinity selection from phage-displayed Ab libraries [46, 55, 73] or transformation of human B cells from HCV infected individuals [44, 72, 74]. Neutralizing murine mAbs to this region has also been isolated following immunization with E2 [38]. Interestingly, there is a third class of Abs targeting this region typified by a human mAb isolated from a phage display library, e137 [52, 75]. This mAb has a unique epitope specificity that overlaps both the 412–423 and the 523–535 neutralizing regions. Key contact residues are T416, W420, W529, G530, and D535, suggesting that the AR3 and the epitope cluster typified by AP33 are proximal on the surface of the E2 protein associated with virions [52]. Another unique epitope overlapping the CD81 binding site has also recently been described. Immunization of an alpaca with recombinant E2 resulted in the production of anti-E2 heavy chain-only Abs (HCAbs) with specificity for the E2 ectodomain [76]. Cloning of the antigen binding domains of these Abs (nanobodies) identified nanobody D03 that possessed epitope specificity for the conserved amino acids N415, G523, and T526. This nanobody was able to both neutralize infectivity and prevent cell-to-cell transmission of HCV. Together these data suggest that the CD81 binding site is a promising target for administration of therapeutic Abs. It is highly conserved [19] and there is an absolute requirement for CD81 binding for entry all strains so far described [5, 9, 21, 77]. This region is also resistant to the emergence of escape mutations [42], indicating that therapy might not suffer from short-term efficacy.

### 3.4. Other Epitopes in E2

In addition to this major antigenic region overlapping the CD81 binding site, other antigenic domains have also been defined in independent studies by reactivity to human mAbs. A region containing a type-restricted neutralization epitope (AR2) closely overlaps the “Antigenic Domain C,” defined by the mAbs CBH-7 and AR2A, centred on an asparagine residue at position 540 [46, 72]. While this epitope region is not extensively characterized, competition assays revealed that this epitope is discrete from those recognized by murine conformation-independent mAbs. The restricted neutralization observed by CBH-7 and AR2A suggests that amino acids positions tolerant to change are the target of these Abs. From this it can be inferred that these regions of E2 are unlikely to play a key role in the entry cascade and therefore are unlikely to be a good choice to pursue for therapeutic intervention. An additional antigenic region, AR1, has also been identified, with contact residues around V538 and N540. This too overlaps with the epitope of CBH-7 [46] but has no neutralizing activity. It is striking that despite the differences in phenotype between Abs representing different antigenic regions, their epitopes all appear to cluster around a small area of the surface of E2, in the proposed DI [23]. Structural analysis is required to elucidate if this is the case, or whether the common competition with CBH-7 is due to steric hindrance.

Recently two additional antigenic regions have been proposed. Having been well established that the CD81 binding site is the most likely target of neutralizing Abs isolated from human Ab libraries, Giang and colleagues pre-blocked the E2 glycoprotein with known CD81-binding site Abs before selecting a phage-displayed Ab library for affinity to the protein complex [26, 78]. This yielded novel Abs with specificity for epitopes outside the CD81 binding site, binding to regions defined as “Antigenic Region 4 and 5” (AR4 and AR5). Notably, mAb AR4A potently neutralized a range of genetically diverse strains and was able to limit infection *in vivo *[26]. Binding of AR4A and AR5A was dependent on the presence of E1 as well as E2, suggesting that the conformation of the heterodimer is important to their epitopes. Indeed, some mutations in E1 affected binding of both of these Abs. However, distinct residues in E2 were found to be important for each of these Abs, with D698 being essential for AR4A binding, while R639 was essential for mAb AR5A binding (Table 1). These epitopes are located in an otherwise undescribed cluster in a membrane proximal region in E2. However, in common with AR1, AR2 and AR3, AR5A were observed to compete with mAb CBH-7, placing the epitope of this human mAb in a central position overlapping the different antigenic faces of E2. In contrast, AR4A did not compete with CBH-7, emphasizing the unique nature of this Ab epitope.

## 4. Nonneutralizing/Interfering Anti-HCV/E2 Abs and Topography of Targeted Epitopes

### 4.1. The Concept of Ab-Mediated Interference

The concept of nonneutralizing/interfering Abs elicitation by pathogens is historically well known. It was first hypothesized by Dulbecco et al. in 1956 analyzing the Ab-mediated neutralization of two animal viruses (Western equine encephalomyelitis and poliomyelitis type 1). In particular, they had observed an inhibition of virus neutralization exerted by some serum samples that probably contained Abs able to inhibit the binding of neutralizing Abs without affecting virus infectivity [79]. 

In the following years, this phenomenon has acquired a gradually relevant interest giving its possible role in the establishment of chronic viral infections, in which the virus persists notwithstanding the presence of an excess of neutralizing Abs. In particular, very recently, its role is widely debated for HCV and other hypervariable viruses (such as Influenza and HIV) as a further escape mechanism to continually overcome the humoral immune response of the host [80–86]. 

At present mAbs represent the best available tools to study the role of nonneutralizing/interfering B-cell epitopes, giving their standardized behavior compared to polyclonal preparations (i.e., specific reactivity with individual antigenic determinants) and indeed the ideal probes for studying the spatial relationship or topography of viral targeted epitopes and their role in neutralization [87].

### 4.2. Role of Nonneutralizing/Interfering Abs in HCV Infection

The hypothesis of the mechanism of Ab-mediated interference in HCV infection was firstly investigated using anti-HCV/E2 mAbs isolated from a chronically HCV-infected patient [88]. In particular, we observed that binding of monoclonal Fabs to HCV/E2 caused conformational changes modifying Fab-binding patterns and reducing, with a negative synergistic effect, Fab-mediated neutralization of binding (NOB) activity to the CD81 receptor. Indeed, our study hypothesized that some Ab clones have the potential to modify HCV/E2 conformation and that, in this state, binding of this glycoprotein to its cellular target is less prone to inhibition by some Ab clones.

More recently, a paper by Zhang et al. finely demonstrated our hypothesis and mapped the HCV/E2 B-cell epitopes involved [89]. In particular, they observed that neutralizing Abs, directed against an E2 region involved in HCV binding to CD81, and in particular encompassing epitope I, could be hindered by the presence of nonneutralizing Abs which bind residues within epitope II, spanning amino acid residues 434–446. Interestingly, they observed that blocking or deletion of these interfering epitope II-specific Abs not only raised the neutralizing titer of HCV-positive sera containing both epitope I- and epitope II-specific Abs but also uncovered a broader cross-genotype neutralizing response. 

Recently, we have confirmed the observation of Zhang et al. using anti-HCV/E2 mAbs and analyzing their activity *in vitro* through HCVcc and HCVpp neutralization assays [50]. In particular, it has been found that among the mAbs isolated from a chronically HCV-infected patient, the human mAb e509, recognizing residues within epitope II, was able to interfere with the broadly neutralizing activity of the mouse mAb AP33, which is known to bind the epitope I region. In particular, competition assays suggest that the binding of e509 interferes with AP33 activity by sterically hindering its binding to its epitope or possibly inducing conformational changes on E2 that inhibit AP33 interaction. 

On the other hand, the activity of two other broadly cross-neutralizing mAbs, e20 and e137, was not minimally affected by e509. One of the reasons for this lack of interference is probably that e20 and e137 mostly bind residues targeted by neutralizing Abs in another CD81-binding region of HCV/E2 (in particular that encompassing amino acid residues 529–535) outside epitope I and therefore potentially less subjected to the interfering effect of epitope II-directed Abs. Moreover, in contrast to AP33, e20 and in particular e137 also bind residues within the interfering epitope II, but at higher affinity than e509, thus displacing it from HCV/E2. In fact, as speculated by Duan et al. and also recently confirmed in the work by Keck et al., the neutralization ability appears to be correlated with the overall binding affinity of these mAbs to epitope II [40, 47]. Furthermore, similar to e20 and e137, it has been described that some mAbs which bind the antigenic domain B of HCV/E2 also contain residues located within epitope II (Table 1) [42]. 

In contrast to these findings, we recently observed that different Abs targeting the region encompassing epitope II not only neutralized HCVpp and HCVcc infection but augmented neutralization mediated by Abs targeting the region encompassing epitope I. In particular, for these studies, the murine and rat mAbs AP33 and 2/69a (with the last one targeting epitope II), as well as human immunoglobulin fractions affinity purified on linear peptides representing distinct HCV/E2 domains clustering within the regions 412–426 and 434–446, were used. Combining these Abs together, we failed to demonstrate any inhibition between these two groups of Abs [37]. These results provided evidence that interference by nonneutralizing Abs, at least to the region encompassing residues 434–446, is not a mechanism for HCV persistence in chronically infected individuals, as it had been originally proposed by Zhang and colleagues.

However, this study confirmed, as previously observed by other groups and by patient sera analysis of binding to epitope I and epitope II, that these two regions are co-immunogenic despite being both recognized by the sera of only a small subset of patients [35, 89]. Indeed, this observation further confirms the known low immunogenicity of the epitope I region compared to epitope II, considering also the less conserved nature of this last epitope [35]. Furthermore, these data confirm the mostly conformational nature of epitope II as previously suggested by our data and by previous structural studies [23, 90]. In fact, as expected, binding assays performed on peptides spanning conformational regions could not completely predict their immunogenicity as Abs directed against conformational epitopes could not be detected. Indeed, we found that, depending on the infected individual, Abs targeting the region encompassing epitope II could differ in phenotype, according to their epitope specificity, or could exhibit a dual phenotype [37].

In accordance with these findings, Keck et al. described anti-HCV/E2 human mAbs binding conformation-sensitive epitopes encompassing also some residues within the 434–446 interfering region. These mAbs are broadly neutralizing and do not lead to viral escape mutants, demonstrating the functional importance of their epitopes. The authors conclude that not all Abs directed against epitope II are interfering and that this activity could be limited to Abs recognizing linear epitopes within it [58]. 

Furthermore, in a more recent work, Keck et al. isolated human mAbs (named HC33) directed against a peptide spanning epitope I region possessing varying neutralizing activity against HCVcc of genotype 1–6, depending on their affinity for the antigen. Moreover, they observed a unidirectional competition for binding to E2 between HC33 mAbs and human mAbs to the 434–446 amino acid region. In addition, when neutralizing HC33 mAbs were combined with HC-11 or HC84 mAbs (whose epitopes encompass the proposed “Antigenic Domain D” and have contact residues located within the epitope II), they observed antagonistic effect at lower Ab concentrations and synergistic effect at higher Ab concentrations both in neutralization and NOB assays [47]. 

It is noteworthy that another recent paper supported the possibility of Ab mediated interference in *in vivo *experiments. In particular, treating an HCV chronically infected chimpanzee using HCV1 mAb, the authors observed an interfering effect, probably Ab mediated, of the chimpanzee serum on the neutralizing activity of HCV1. In fact, similar to AP33, HCV1 recognizes the epitope I region of E2 with a comparable affinity [91].

Furthermore, recent clinical trials on HCV genotype 1a-infected patients undergoing liver transplantation evidenced a viral rebound after 28 and 2 days after transplantation in mAb HCV1-treated patients and placebo-treated patients, respectively. The authors observed the occurrence of viral variants within the epitope I region that could determine escape from HCV1 neutralization [92]. However, possible patient-interfering Abs could negatively influence the neutralizing activity of the mAb, but the authors did not investigate on this possible escape mechanism. 

### 4.3. Topography of HCV/E2 Epitope II

As anticipated, differently from the highly conserved nature of epitope I, epitope II is less conserved as it is located within the third hypervariable region of E2 (HVR3), which encompasses also another neutralizing CD81 binding region, suggesting the conformation-sensitive nature of epitope II [18]. 

In this regard, Duan et al. finely mapped the amino acid residues of epitope II bound by nonneutralizing/interfering Abs. In particular they isolated four mouse mAbs, two of which (#8 and #41) were able to neutralize *in vitro* HCV of genotype 1a, while the other two (#12 and #50) failed to neutralize the virus. Interestingly, #12 mAb could interfere with the neutralizing activity of a chimpanzee polyclonal Ab and of a specific human immunoglobulin preparation both directed against epitope I of HCV/E2. The authors predicted indeed that residues W437, L438, and L441 (located within epitope II) were the common direct contact points for the binding of #8, #41, #12, and #50 mAbs. Interestingly, #12 and #50 binding was more affected by substitution at L441 and F442 than #8 and #41, suggesting that these residues may be more related to the binding of nonneutralizing Abs rather than that of neutralizing Abs (Table 1) [40]. Moreover, in a recent work Deng et al., attempting to better define at the atomic level the fundamental mechanism of Ab-mediated neutralization, reported the crystal structure of the epitope II peptide in complex with mAb #8. In particular, this group found that mAb #8 interacts both with the C-terminal *α*-helix (contacting aminoacid residues W437 and L438) and the N-terminal loop (contacting aminoacid residues E431 and N434) of epitope II. Indeed the authors speculated that neutralization may be achieved through bifurcated Ab-binding to these two regions, compared to nonneutralizing Abs which simply bind only *α*-helical structure of epitope II [41].

In this regard, conducting a meta-analysis of the data reported by Duan et al., we can observe that substitutions at W437 and F442 affected also e20, e137, and e509 binding, while substitutions at L438 and L441 affected only e137 and e509 binding, again confirming the speculations of the authors that both L441 and F442 are determinant for the binding of nonneutralizing and interfering Abs [40, 49, 50, 52]. 

Finally, the predicted recognition of W437 by e20 and e137 does not limit their cross-recognition and cross-neutralization profile of different genotypes/isolates as conversely observed by Duan et al. [40]. Further analysis on the binding of these two cross-neutralizing mAbs to the N-terminal loop of epitope II must be conducted. However, similar to other previously described broadly neutralizing mAbs, the neutralization mechanism of e20 and e137 appear to be mediated by recognition of different discontinuous conformational epitopes involving amino acid regions 412–424, 436–447, and 523–540 of HCV/E2 glycoprotein [42, 52]. In fact, as suggested by Lapierre et al., broadly neutralizing activity of these mAbs is the result of the recognition of structural determinants rather than specific residues of the conformational epitope II [90]. These speculations raise the possibility that residues L441 and F442 are both contact residues only for e509 and thus confirming that the sequence 441-LFY-443 was linked to the non-neutralization of the virus [40].

However, the role (and the existence itself) of interfering Abs in influencing HCV infection is still controversial and, as concluded by the authors themselves, caution must be taken in differentiating neutralizing Abs from nonneutralizing Abs solely on the basis of their residue specificity. Indeed, minor change of residues in the Ab-antigen interface, epitope frameshifting among genotypes/isolates, as well as change in binding affinity may alter recognition capabilities of these Abs and thereby may consequently modulate their activity [40]. 

Interestingly, according to the putative model for E2 folding, all the three aforementioned regions would lie next to each other on the glycoprotein [23]. Therefore, this structural prediction possibly supports the interfering effect of epitope II-directed Abs. However while this predicted structure is currently the best model available, these conclusions cannot be absolutely asserted. For this purpose, the availability of E1-E2 crystal will certainly accelerate the fine elucidation of the spatial proximities of neutralizing and interfering epitopes on the E1-E2 structure and, consequently, structure-based vaccine progress.

Finally, the low prevalence and the low titer of epitope I-reactive Abs in sera from both chronic and acute resolved infections further support the hypothesis of a conformational masking by adjacent regions such as epitope II [71, 89]. In fact, Zhang et al. originally put forward the idea that once epitope II is bound to an Ab, the site of epitope I becomes masked and can no longer be recognized by specific neutralizing Abs. Indeed, depletion of Abs to epitope II in plasma from a chronically infected HCV patient and vaccinated chimpanzees recovered an otherwise undetectable cross-genotype neutralizing activity [89]. Another possibility is that the initial binding of interfering Abs to the region containing epitope II may induce conformational changes on E2 that inhibit the binding by epitope I-directed Abs, as recently suggested by Lapierre et al. for other anti-HCV/E2 Abs [90]. 

Thus, the described divergent observations reported above may depend on the different Ab specificities present in the polyclonal preparations used and, probably, also on the different HCV genotypes infecting the considered cohort of patients. Moreover, the different strategies adopted in isolating epitope I- and epitope II-directed Abs could explain the different data obtained. In fact, immunoglobulins purified on peptides representing distinct HCV/E2 regions are obviously directed against linear epitopes; these preparations are certainly different from mAbs cloned using a full-length HCV/E2 glycoprotein, which are more probably directed against conformational epitopes including also residues outside the investigated linear regions. 

To summarize, in the HCV field several works support the existence of interfering Ab populations and hypothesize their possible role in HCV persistence, as demonstrated using different Ab preparations such as human plasma-derived immunoglobulin preparations, human mAbs, and sera of animals immunized with recombinant HCV/E2 peptides. The possible mechanism leading to the interference is still controversial, but both direct steric-hindrance and induced antigen conformational changes have been hypothesized. On the other hand, other papers do not confirm these findings, suggesting that the putative interfering epitope II may be targeted by Abs endowed with a broadly neutralizing activity. However other studies suggest that the interfering Abs do exist but that their overall effect may be biased by the presence of neutralizing Abs with different binding features and by the infecting HCV genotype. Future works further investigating the *in vivo *role of these interfering Ab subpopulations in HCV persistence are certainly needed.

## 5. Conclusions

Although much of the research into neutralizing epitopes has focused on the E2 glycoprotein, some neutralizing determinants have been identified also in E1. Efforts to identify anti-E1 Abs have been hindered by the poor folding of E1 when expressed *in vitro *[93]. Despite this, mAbs directed to E1 192–211 amino acid region have been identified to be cross-reactive with E1 samples representing multiple genotypes and neutralize entry [94], while neutralizing mAbs IGH505 and IGH526 recognize an epitope between the 313–327 amino acid region of E1 and neutralize entry of diverse isolates [95]. However, immunization with E1 alone does not induce a neutralizing response in experimental animals [53]. Although E1 is an interesting target for discovery of neutralizing Abs, the current paucity of Abs directed to this protein limits greater understanding of its antigenic structure.

Furthermore, the escape mechanism utilizing the elicitation of nonneutralizing Ab subpopulations able to enhance the viral infectivity or able to interfere with the activity of neutralizing Abs has further implications in vaccine design as well as in the development of effective therapeutic mAbs [83]. Indeed, many anti-HCV/E2 mAbs recently described in the literature as well as those currently undergoing clinical trials target regions that could potentially be subjected to the Ab-mediated interfering mechanism [92]. Moreover, other mechanisms of HCV escape from the humoral immune response must be taken into account when considering the potential clinical applications of developing antiviral mAbs such as the lipoprotein and glycan shielding of the viral envelope as well as the recently described glycan shifting consequent to the high mutation rate of the virus [36], the main reason for incomplete effectiveness of currently available therapies as well as the main hurdle for candidate therapeutics under development. Finally, the observed cell-to-cell route of infection for HCV may further limit the breadth of viral entry inhibitors (e.g., anti-E1 and anti-E2 mAbs) in particular in those chronically infected patients undergoing liver transplantation with the consequent augmented risk of graft reinfection and indeed of a more progressive and complicated disease [96]. However, Abs able to neutralize this route of transmission have been recently described [76]. On the other hand, a further therapeutic approach overwhelming these escape mechanisms would consist in the administration of entry inhibitors together with other antiviral compounds targeting different steps of the viral replication cycle such as protease and polymerase inhibitors [97, 98]. In fact, it is well known that in the treatment of chronic diseases caused by hypervariable infectious agents, the administration of single or multiple drugs targeting only limited step of their life cycle leads, in the majority of cases, to the occurrence of escape variants limiting their effectiveness that could be further complicated by an incomplete adherence of the patients to the therapeutic regimen. In this regard, the occurrence of viral variants after treatment of HCV chronic infections with the newly introduced protease inhibitors (Boceprevir and Telaprevir), whose administration is indicated only for genotype 1 infected patients, has been well demonstrated [99]. However, new more effective antiviral drugs targeting the NS3 protein and other viral proteins as well as other cellular targets implicated in the HCV viral life cycle, such as the CD81 receptor, are under development and clinical evaluation [100]. Interestingly, anti-CD81 compounds have shown to limit the cell-to-cell spread of HCV and thus represent valuable candidates for a future combined therapy [101]. Moreover, differently from currently available therapies, the targeting of cellular determinants, such as CD81, may be theoretically beneficial for patients infected with any HCV genotype and could be less subjected to the occurrence of viral variants. Thus, the HCV entry inhibition by antivirals directed against viral and cellular determinants could be an effective and valuable tool, in particular in the course of a post-transplant setting, for which no therapies are currently available.

## Figures and Tables

**Table 1:** Schematic representation of the regions targeted by anti-HCV/E2 mAbs. Involved domains (DI, DII and DIII as well as the stem region) of HCV/E2 have been evidenced. In particular, DI has been described to be a discontinuous region containing the CD81 binding site; DII is predicted to possess the fusion peptide and DIII has been recently described to contain antigenic neutralization epitopes and to be involved in heterodimerization with E1.

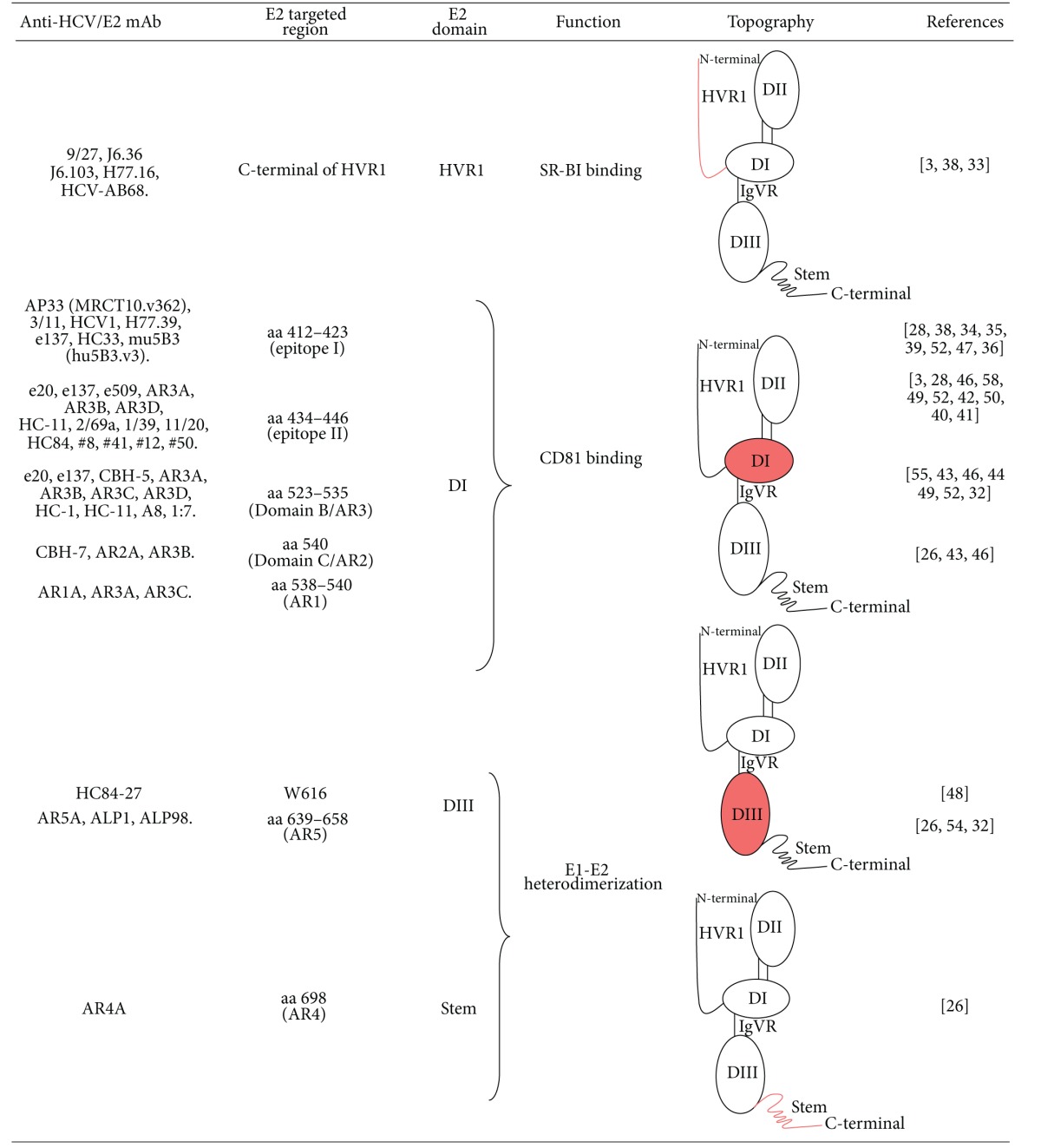

**Table 2:** Synopsis of broadly anti-HCV/E2 neutralizing mAbs.

mAb	Origin	Epitope	Genotype breadth of neutralization (HCVpp)	Genotype breadth of neutralization (HCVcc)	*In vivo *tested	Escape generation	References
9/27	Rat	Linear	1a	1a, 2a	No	N.D.	[3, 28, 32]
HCV-AB68	Human	Conformational	1b	N.D.	Yes	Yes	[33]
AP33 (MRCT10.v362)	Murine (humanized)	Linear	1–6	1a, 2a	Yes	Yes	[34–36]
3/11	Rat	Linear	1, 2, 4, 5, 6	1a, 2a	No	N.D.	[3, 28]
2/69a	Rat	Conformational	1a, 2a	1a	No	N.D.	[3, 28, 37]
11/20	Rat	Conformational	1	N.D.	No	N.D.	[3, 28]
1/39	Rat	Conformational	1	N.D.	No	N.D.	
H77.16	Murine	Conformational	N.D.	1a	No	N.D.	[38]
H77.39	Murine	Conformational	N.D.	1a	No	N.D.	
J6.36	Murine	Conformational	N.D.	2a	No	N.D.	
J6.103	Murine	Conformational	N.D.	2a	No	N.D.	
HCV1	Human	Linear	1a, 1b, 2b, 3a, 4a	2a	Yes	Yes	[39]
#8	Murine	Conformational	N.D.	1a	No	N.D.	[40, 41]
HC-1	Human	Conformational	1a, 1b	2a	No	No	[42]
HC-11	Human	Conformational	N.D.	2a	No	Yes	
CBH-5	Human	Conformational	1–6	2a, 2b	No	N.D.	[43, 44]
CBH-7	Human	Conformational	1, 2, 4	2a, 2b	No	N.D.	
A8	Human	Conformational	1–6	2a	No	N.D.	[45]
1:7	Human	Conformational	1–6	2a	No	N.D.	
AR2A	Human	Conformational	1a, 2a, 2b, 4, 5	No	No	N.D.	[46]
AR3A	Human	Conformational	1–6	1–6	Yes	N.D.	[26, 46]
AR3B	Human	Conformational	1a, 1b, 2a, 2b, 4, 5	2a	Yes	N.D.	[46]
AR3C	Human	Conformational	1a, 1b, 2a, 2b, 4, 5	2a	No	N.D.	
AR3D	Human	Conformational	1a, 1b, 2a, 2b, 4, 5	2a	No	N.D.	
AR4A	Human	Conformational	1–6	1–6	Yes	N.D.	[26]
AR5A	Human	Conformational	1a, 1b, 4, 5, 6	1a, 2a, 4, 5, 6	No	ND	
HC33.1	Human	Linear	N.D.	1a–6a	No	N.D.	[47]
HC33.4	Human	Linear	N.D.	1a–6a	No	N.D.	
HC33.8	Human	Linear	N.D.	1a, 2a, 4a	No	N.D.	
HC33.29	Human	Linear	N.D.	1a, 2a, 4a	No	N.D.	
mu5B3 (hu5B3.v3)	Murine (humanized)	Linear	1a, 1b, 2a	2a	No	Yes	[36]
HC84-1	Human	Conformational	1a	1a–6a	No	No	[48]
HC84-27	Human	Conformational	1a	1a–6a	No	No	
e20	Human	Conformational	1a, 1b, 2a, 2b, 4, 5 (N.D. on 3 and 6)	1a, 2a	No	N.D.	[49–51]
e137	Human	Conformational	1a, 1b, 2a, 2b, 4, 5 (N.D. on 3 and 6)	1a, 2a	No	N.D.	[50–52]

ND: not determined.
